# Non-invasive diagnosis and staging of non-alcoholic fatty liver disease

**DOI:** 10.1007/s42000-022-00377-8

**Published:** 2022-06-04

**Authors:** Stergios Kechagias, Mattias Ekstedt, Christian Simonsson, Patrik Nasr

**Affiliations:** 1 Department of Gastroenterology and Hepatology, University Hospital, Linköping, Sweden; 2 Department of Health, Medical and Caring Sciences, Linköping University, Linköping, Sweden; 3 Center for Medical Image Science and Visualization (CMIV), Linköping University, Linköping, Sweden; 4 Department of Biomedical Engineering, Linköping University, Linköping, Sweden

**Keywords:** Fibrosis, Non-alcoholic fatty liver disease, Non-invasive tests, Steatosis

## Abstract

Non-alcoholic fatty liver disease (NAFLD) is considered to be the hepatic manifestation of the metabolic syndrome and is characterized by ectopic accumulation of triglycerides in the cytoplasm of hepatocytes, i.e., steatosis. NAFLD has become the most common chronic liver disease, with an estimated global prevalence of 25%. Although the majority of NAFLD patients will never experience liver-related complications, the progressive potential of NAFLD is indisputable, with 5–10% of subjects progressing to cirrhosis, end-stage liver disease, or hepatocellular carcinoma. NAFLD patients with advanced fibrosis are at the highest risk of developing cardiovascular and cirrhosis-related complications. Liver biopsy has hitherto been considered the reference method for evaluation of hepatic steatosis and fibrosis stage. Given the limitations of biopsy for widescale screening, non-invasive tests (NITs) for assessment of steatosis and fibrosis stage, including serum-based algorithms and ultrasound- and magnetic resonance-based methods, will play an increasing role in the management of NAFLD patients. This comprehensive review presents the advantages and limitations of NITs for identification of steatosis and advanced fibrosis in NAFLD. The clinical implications of using NITs to identify and manage NAFLD patients are also discussed.

## Introduction

**Fig. 1:**
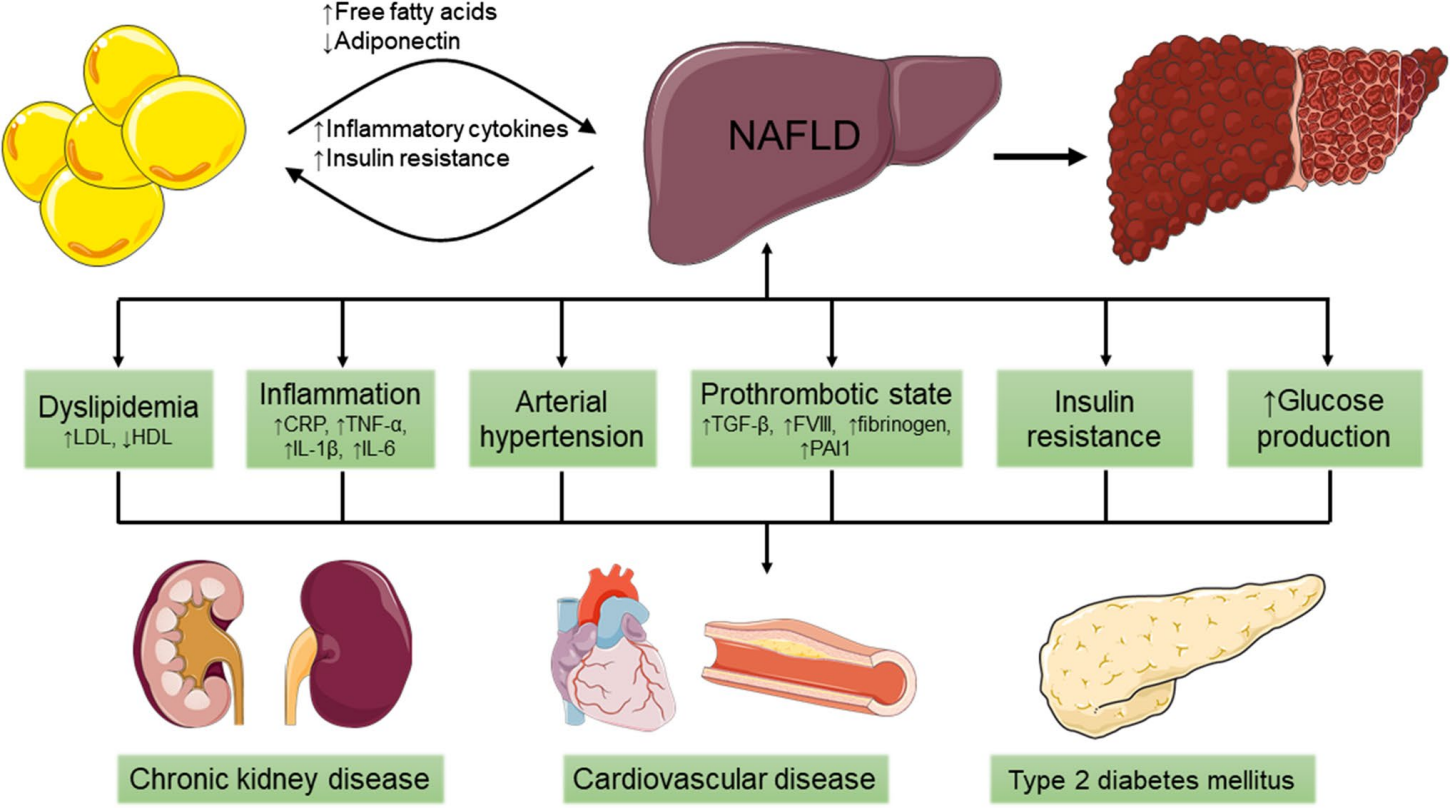
NAFLD is associated with an increased risk of developing chronic kidney disease, cardiovascular disease, and type 2 diabetes mellitus. There are several putative mechanisms underlying this association. NAFLD contributes to an atherogenic milieu (dyslipidemia with decreased HDL and increased LDL) and a prothrombotic state (increased levels of TGF-β, fibrinogen, factor VIII, and PAI-1), as well as arterial hypertension, hepatic and systemic insulin resistance, increased glucose production, and increased secretion of proinflammatory biomarkers (such as CRP, IL6, IL-1β, and TNF-α). Furthermore, several of these pathological mechanisms seem to have a bidirectional effect, both increasing the presence of NAFLD and consequently increasing the risk of NAFLD progression to NASH and cirrhosis. Images from Servier Medical Art (https://smart.servier.com/). Abbreviations: CRP, C-reactive protein; FVIII, factor VIII; HDL, high-density lipoprotein; IL-1β, interleukin-1β; IL-6, interleukin-6; LDL, low-density lipoprotein; NAFLD, non-alcoholic fatty liver disease; NASH, non-alcoholic steatohepatitis; PAI-1, plasminogen activator inhibitor-1; TGF-β, transforming growth factor-β; TNF-α, tumor necrosis factor-α

Non-alcoholic fatty liver disease (NAFLD) affects approximately 25% of the global population [1] and has in recent years surpassed viral hepatitis as a major cause of chronic liver disease [2, 3]. NAFLD is the hepatic manifestation of the metabolic syndrome and encompasses a spectrum of histopathological features that range from ectopic accumulation of triglycerides in the cytoplasm of hepatocytes (i.e., steatosis) via establishment of inflammation and hepatocellular injury (i.e*.*, non-alcoholic steatohepatitis (NASH)), to progressive fibrosis and risk of progression to cirrhosis and development of end-stage liver disease (ESLD) or hepatocellular carcinoma (HCC) [4, 5]. The presence of NAFLD is associated with type 2 diabetes mellitus (T2DM), as well as with a greater prevalence and incidence of cardiovascular disease and chronic kidney disease [6] (Fig. 1). In patients with T2DM, NAFLD prevalence ranges from 70 to 95%, while the rate is even higher in morbid obesity at up to 98% [1]. Subjects with T2DM are particularly susceptible to more severe forms of NAFLD and its associated consequences [7, 8] as they have a higher prevalence of advanced fibrosis compared to the general population [9]. The swift increase in the prevalence of NAFLD is also mirrored in the incidence of ESLD. In the USA, NAFLD is currently the second leading indication for liver transplantation [10] and the most rapidly growing cause of HCC among subjects listed for liver transplantation [11]. A modeling study suggests that by 2030, the prevalence of NASH will have risen by as much as 56%, with ESLD and mortality expected to more than double [12].

Liver biopsy is considered the reference method for diagnosing hepatic steatosis. Moreover, histologic evaluation of liver tissue enables assessment of the presence of NASH with or without advanced fibrosis. However, liver biopsy sampling represents only approximately 1/50,000 of the organ volume and has its own inherent limitations, such as cost, sampling errors due to inadequate sample acquisition, incorrect sample representation, observer variability, and risk of adverse events, making it unsuitable for large-scale screening [13]. Therefore, simple, easily accessible, and validated non-invasive tests (NITs) are of utmost importance and much needed in the management of NAFLD patients.

Presence of hepatic steatosis is the prerequisite for diagnosing NAFLD [14]. Therefore, this review will first focus on the non-invasive diagnosis of steatosis. Although the severity of steatosis is associated with development of T2DM and mortality [15], hepatic fibrosis has been shown to be the key independent predictor that is associated with all-cause, cardiovascular, and liver-related mortality in NAFLD patients [16–19]. Lobular inflammation and hepatocellular ballooning, included in the histopathological NAFLD activity score (NAS), have not been shown to predict progression of fibrosis or mortality [17, 18]. After the diagnosis of steatosis, the stage of fibrosis should be assessed appropriately. In clinical practice, identification of NAFLD patients with advanced fibrosis (F3-F4) is a priority as these patients are at higher risk of mortality and development of liver-related complications [20]. It is widely accepted that NAFLD patients with advanced fibrosis should be followed up in secondary care and should be prioritized for treatment once medications for NAFLD are recommended. Thus, this review will also focus on NITs to diagnose advanced fibrosis in NAFLD.

The exclusion of other liver diseases, including “significant” alcohol consumption, is necessary to establish a diagnosis of NAFLD. A panel of international experts recently suggested an alternative approach, i.e., “positive criteria” to diagnose the liver disease associated with the metabolic syndrome, while they also proposed the name metabolic dysfunction-associated fatty liver disease (MAFLD) [21]. The criteria are based on evidence of hepatic steatosis (detected either by imaging techniques, blood biomarkers/scores, or liver histology), in addition to one of the following three criteria, namely, overweight/obesity, presence of T2DM, or evidence of metabolic dysregulation. In contrast to NAFLD, MAFLD can coexist with other chronic liver diseases, for example, viral hepatitis or alcohol-related liver disease. To our knowledge, there are to date no large studies that have evaluated the performance of NITs in MAFLD using the proposed definition. Thus, it is currently unknown if the data presented in this review can be extrapolated to MAFLD.

## Steatosis

Hepatic triglyceride content (HTGC) can be assessed using various methods that measure fundamentally different tissue properties. The conventional histopathological methodology used to quantify liver fat content consists of a visual semiquantitative approach in which the histopathologist uses a four-graded scale (0–3). Grades 0–3 are considered to correspond to fat deposition in < 5%, 5–33%, 34–66%, and > 66% of the hepatocytes, respectively [22, 23]. The diagnosis of NAFLD requires that ≥ 5% of the hepatocytes contain fat globules in the absence of overconsumption of alcohol and other (secondary) causes of steatosis. It has previously been demonstrated that semiquantitative assessment of steatosis by a histopathologist frequently overestimates hepatic fat content when measured quantitatively [24]. An alternative approach is to assess steatosis quantitatively with stereological point counting, a method with higher reproducibility suggested as being preferable when accurate histopathological measurements of hepatic steatosis are required [24]. Both the semiquantitative histological method and stereological point counting rely on biopsies and, as such, are invasive tests, which issue will not be further discussed in this review.

### Serum-based steatosis biomarkers

Although levels of alanine aminotransferase (ALT) are the best single biochemical correlate of hepatic steatosis, serum levels of hepatic enzymes can be within reference limits in 50–79% of NAFLD patients, fluctuating or slightly elevated [25, 26]. There have been suggestions that the current upper limit of normal (ULN) for ALT may be too high and should be reduced significantly [27, 28]. Slight to moderate elevation of serum ferritin can be caused by dysmetabolic iron overload associated with steatosis. However, in many NAFLD patients, elevated ferritin may reflect a subclinical inflammatory state rather than iron overload [29]. In general, a single biomarker cannot be used to identify NAFLD patients.

Several panels in which serum-based biomarkers are included have been evaluated as predictors of hepatic steatosis. Among them are fatty liver index (FLI) [30], hepatic steatosis index (HSI) [31], NAFLD liver fat score (NAFLD-LFS) [32], SteatoTest [33], visceral adiposity index (VAI) [34], triglyceride × glucose (TyG) index [35], and lipid accumulation product (LAP) [36, 37]. The diagnostic performance of these tests is summarized in Table 1. It should be noted that several of these tests have been evaluated using ultrasound as the gold standard to diagnose steatosis. However, ultrasound lacks sensitivity for detection of low grades of steatosis (see below) and, thus, the diagnostic performance of HSI, FLI, and LAP has probably been overestimated. In a study in which ultrasonography, SteatoTest, and liver biopsy were performed in 304 patients, it was shown that concordance between steatosis diagnosed both with ultrasonography and histopathologically was lower (kappa coefficient = 0.32 ± 0.05) than the concordance between SteatoTest and biopsy (kappa coefficient = 0.44 ± 0.06; *P* = 0.02) [33], indicating that both ultrasonography and serum-based tests have suboptimal sensitivity in detecting steatosis.

**Table 1:** Serum-based steatosis markers

Blood markers/algorithms	Components or formulas	AUROC	Study population (no. of participants)/diagnostic tools
Hepatic steatosis index[31]	8 × ALT/AST + BMI (+ 2, if type 2 diabetes; + 2, if female)	0.72–0.82	Korean (*n* = 10,724)/ultrasound
Fatty liver index[30]	(e^0.953 × ln (TG) + 0.139*BMI + 0.718 × ln^ ^(GGT) + 0.053 × WC − 15.745^)/(1 + e ^0.953 × ln^ ^(TG) + 0.139 × BMI + 0.718 × ln (GGT) + 0.053 × WC − 15.745^) × 100	0.79–0.85	Italian (*n* = 496)/ultrasound
NAFLD liver fat score[32]	− 2.89 + 1.18 × metabolic syndrome (yes = 1/no = 0) + 0.45 × type 2 diabetes (yes = 2/no = 0) + 0.15 × insulin (mU/L) + 0.04 × AST − 0.94 × AST/ALT	0.78–0.87	Finnish (*n* = 470)/^1^H-MRS
SteatoTest[33]	ALT, α2-macroglobulin, apolipoprotein A1, haptoglobin, total bilirubin, GGT, total cholesterol, TG, glucose, age, gender, BMI	0.72–0.86	Caucasians (*n* = 2272)/liver biopsy Patented test. The model equation was not presented
Lipid accumulation product (LAP)[36, 37]	[WC (cm)–65 (male) or –58 (female)] × [TG (mmol/L)]	0.72–0.83	NHANES III (development, *n* = 9180), Italian (validation, *n* = 588)/ultrasound (in evaluation study) Originally developed as an index of cardiometabolic risk
Visceral adiposity index[34]	Male: [WC/39.68 + (1.88 × BMI)] × (TG/1.03) × (1.31/HDL) Female: [WC/36.58 + (1.89 × BMI) × (TG/0.81) × (1.52/HDL)	0.92	Validated in French NAFLD patients (*n* = 324)/liver biopsy Originally developed in patients with hepatitis C
Triglyceride/glucose index[35]	Log (TG × glucose/2)	0.90	Validated in French NAFLD patients (*n* = 324)/liver biopsy Originally developed as a measure of insulin sensitivity

Abbreviations: *ALT*, alanine aminotransferase; *AST*, aspartate aminotransferase; *AUROC*, area under the receiver operating characteristic; *BMI*, body mass index; *GGT*, gamma glutamyltransferase; *HDL*, high-density lipoprotein; *NAFLD*, non-alcoholic fatty liver disease; *TG*, triglycerides; *WC*, waist circumference

In a head-to-head comparison using liver biopsy as the reference standard, VAI outperformed four other algorithms with an area under the receiver operating characteristic (AUROC) of 0.92, a sensitivity of 79%, specificity of 92%, negative predictive value (NPV) of 16%, and positive predictive value (PPV) of 99% [38].

Given their diagnostic performance, the algorithms may have a role in identifying hepatic steatosis or cardiometabolic risk factors in population-based studies [36], but currently, they cannot be used in clinical practice for the diagnosis of NAFLD or in the decision-making process in the management of NAFLD patients. Further studies are needed to determine which algorithm(s) can be recommended as an initial tool for NAFLD screening. Moreover, it has not yet been clarified if these algorithms can be used for monitoring patients during interventions aiming to reduce liver fat.

### Ultrasonography

B-mode ultrasonography is widely used as the first-line imaging modality to detect hepatic steatosis due to its ease of use and low cost. This modality is the recommended screening method to detect steatosis in patients with T2DM by the European NAFLD guidelines [39]. Assessment of liver-to-kidney contrast, parenchymal brightness, deep beam attenuation, brightness of vessel walls, and gallbladder wall definition are used to diagnose excessive liver fat [40]. However, ultrasonography is hampered by limited sensitivity for detection of mild (< 20%) steatosis [40]. Detection of steatosis may be improved by the combination of various echographic parameters during the examination and technical improvements in equipment, reaching a sensitivity of 80–85% at a liver fat content of ≥ 12.5% [41, 42]. However, in hepatic fat content of 5 to 9%, sensitivity can be as low as 12% [43]. Since the definition of NAFLD includes the presence of ≥ 5% hepatic steatosis on liver biopsy, this means that a considerable number of NAFLD patients with steatosis grade 1 on liver histology will not be identified by ultrasonography. Other limitations of ultrasonography include its inability to quantify liver fat and its subjectivity and examiner-dependent characteristics, which limit inter- and intraobserver reproducibility [44].

### Controlled attenuation parameter

During the last few years, several techniques based on ultrasonography have been developed, which provide an improved assessment of liver fat, as compared to conventional B-mode ultrasonography, by implementing quantitative approaches. Among them are ultrasound-guided attenuation parameter (UGAP) [45], attenuation imaging (ATI) [46], attenuation coefficient (ATT) [47], and controlled attenuation parameter (CAP), which quantify liver fat by measuring the attenuation of radiofrequency and are based on the principle that echo attenuation is larger in liver tissue with any grade of hepatic steatosis than in the normal liver. The number of studies evaluating UGAP, ATI, and ATT is limited. CAP can be measured simultaneously with liver stiffness measurement (LSM) by vibration-controlled transient elastography (VCTE) and is widely used to assess hepatic steatosis [48]. The diagnostic accuracy of CAP has been validated extensively. A meta-analysis that compared histologically graded steatosis with CAP and included 2375 patients from 19 studies reported AUROCs of 0.82 with a CAP threshold of 248 dB/m for steatosis of > 11%, 0.86 with 268 dB/m for steatosis of > 33%, and 0.89 with 280 dB/m for steatosis of > 66% [49]. However, the CAP value is affected by the presence of obesity and diabetes mellitus [48]. In NAFLD patients, the optimal thresholds for detecting magnetic resonance imaging proton density fat fraction (MRI-PDFF) of ≥ 5% and ≥ 10% were 288 dB/m and 306 dB/m, respectively, which were considerably higher than the optimal CAP thresholds obtained from a meta-analysis with multiple etiologies of liver disease [50]. In another meta-analysis including 2346 patients with chronic liver diseases, it was demonstrated that the accuracy of CAP in detecting histological liver fat > 5% was fair (AUROC of 0.819) [51]. CAP has a high interobserver reproducibility of 0.82 [52], this feature giving it a clear advantage over conventional B-mode ultrasonography. One disadvantage though with CAP is its high rate of measurement failure (0–24%), particularly in obese patients [53]. To overcome this shortcoming, an obesity-specific probe (known as the XL probe) has been developed [54]. However, in NAFLD patients, the threshold for detecting steatosis seems to be higher with the XL probe compared with the conventional probe (known as the M probe) [55]. Thus, the optimal thresholds for detecting steatosis with the different probes remain to be clarified.

### Computed tomography

The radiodensity of different tissues can be assessed with computed tomography (CT). The radiodensity of water is 0 Hounsfield units (HUs) by definition, and air is defined as –1,000 HUs [56]. In non-contrast CT, normal liver parenchyma is approximately 50 to 60 HUs, while fat is –20 to − 100 HUs. Due to inconsistency in HU calibration by external factors, “fat-free spleen” can be used as an internal reference [56]. Hepatic HU < 40 has been suggested as a cut-off value for steatosis (> 30%) [57], while a liver HU–spleen HU value less than − 9 to − 10 can be used as a reference to detect steatosis [57–59]. However, as with ultrasonography, CT has limited sensitivity in detecting mild steatosis (< 30% liver fat) and is also limited by radiation exposure [56, 57]. Thus, CT cannot be recommended as a primary diagnostic tool to detect steatosis.

### Magnetic resonance-based techniques

Proton magnetic resonance spectroscopy (^1^H-MRS) is widely considered the most accurate non-invasive method with which to measure HTGC [60]. The technique identifies signals from protons associated with triglycerides by their resonance frequencies. The fat fraction is given as the fat signal divided by the sum of the water and fat signals [57]. An excellent correlation has been shown between ^1^H-MRS and total lipid quantification in specimens of liver tissue, and it has been suggested that ^1^H-MRS can replace liver biopsy for the assessment of liver fat content [61]. Compared to liver biopsy, a much larger volume (2 × 2 × 2 or 3 × 3 × 3 cm) of liver tissue is assessed, minimizing the likelihood of sampling error. However, ^1^H-MRS remains primarily a research tool due to its low availability and limited clinical application [57, 62].

**Fig. 2:**
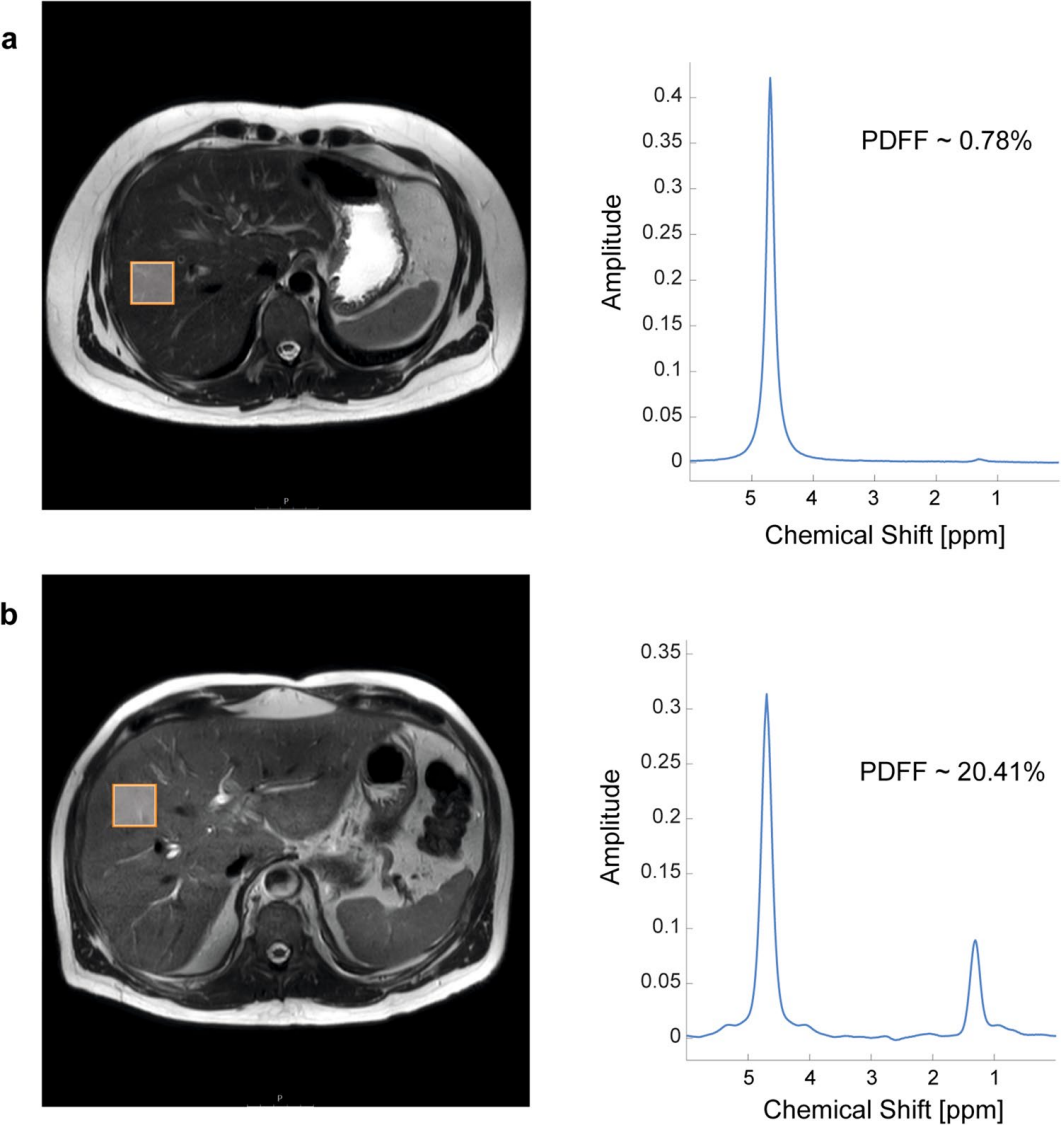
T2-weighted images (left) with schematic placement of ^1^H-MRS voxels (3 × 3 × 3 cm) placed in the right hepatic lobe and corresponding in vivo MRS spectra (right) for water (peak, 4.7 ppm) and triglycerides (peak, 1.3 ppm) for calculation of proton density fat fraction (PDFF) in a subject with **a** normal liver and in a patient with **b** NAFLD. Abbreviations: ^1^H-MRS, proton magnetic resonance spectroscopy; PDFF, proton density fat fraction; ppm, parts per million

In contrast to MRS, magnetic resonance imaging (MRI) is available in many centers. MRI-PDFF is defined as the ratio of the mobile proton density from triglycerides and the total mobile proton density from triglycerides and water and reflects the concentration of triglycerides within liver tissue (Fig. 2). Although MRI-PDFF and histological assessment of fat content measure different properties, studies have demonstrated strong correlations between liver fat quantified by MRI-PDFF and steatosis grade as assessed by liver histology [43, 57]. Bannas et al. assessed the accuracy of MRI-PDFF through linear regression with ^1^H-MRS, triglyceride extraction, and histology using ex vivo human livers [63]. MRI-PDFF showed an excellent correlation with ^1^H-MRS (*r* = 0.984) and a strong correlation with histology (*r* = 0.850) and tissue triglyceride extraction (*r* = 0.871).

In a recent meta-analysis based on six studies including 635 patients with biopsy-proven NAFLD [64], the AUROC value of MRI-PDFF for detecting steatosis was 0.98. Pooled sensitivity and specificity were 93 and 94%. The diagnostic accuracy of MRI-PDFF has been compared with that of CAP in NAFLD patients. MRI-PDFF was superior when a liver biopsy was used as the reference standard [65, 66]. Moreover, in contrast to CAP, the diagnostic performance of MRI-PDFF to diagnose steatosis is not lower in NAFLD patients [64]. An additional advantage of MRI-PDFF is that, unlike ultrasound-based techniques, it can reliably detect longitudinal changes in hepatic triglyceride content as small as 2% or less [67].

In studies and clinical practice using ^1^H-MRS or MRI-PDFF to quantify liver fat content, a cut-off of 5% [68] or 5.56% is usually used to define hepatic steatosis. The latter value is based on the results of Szczepaniak et al. who examined the distribution of HTGC with ^1^H-MRS in 345 subjects with a low risk for hepatic steatosis, i.e., BMI < 25 kg/m^2^, no glucose intolerance or excessive alcohol consumption, and normal serum ALT [62]. However, hepatic steatosis can be present in a significant proportion of lean subjects [69], as well as in individuals with normal serum ALT [70]. Nasr et al. [71] used histopathology as the reference standard to define the optimal cut-off for the definition of hepatic steatosis with ^1^H-MRS and reported that a cut-off of 3.02% could reliably identify patients with steatosis (specificity of 100%). A cut-off of 2% yielded the most reliable diagnostic accuracy, albeit with a few false positives (sensitivity of 87% and specificity of 94%). Thus, the cut-off for identifying NAFLD patients with MRI/MRS-based techniques can reliably be reduced to at least 3%. Other evidence also suggests that 5% as a limit for normal HTGC is too high. For example, it has been reported that a decrease in the suppression of endogenous glucose production is evident with HTGC as low as > 1.5% and that multiple extrahepatic metabolic changes, such as muscle insulin resistance, hypertriglyceridemia, and decreased plasma high-density lipoprotein cholesterol, are fully established at HTGC 6% [25, 41, 72, 73].

An overview of imaging techniques to diagnose steatosis is given in Table 2.

**Table 2:** Imaging techniques to diagnose steatosis

Modality	Description	Diagnostic performance	Advantages	Limitations
Ultrasonography	-Tissue echogenicity depends on the degree of beam scattering -Fat deposition in tissue accentuates scattering	AUROC 0.93 (Sn 60–80%, Sp 80–100%)	-Widely available -Low cost -No radiation -Easy to perform	-Low sensitivity for mild steatosis -Operator-dependent -Reduction of Sn and Sp in obese patients
Controlled attenuation parameter (CAP)	-Measurement of the degree of ultrasound attenuation by hepatic fat	AUROC 0.82 (Sn 69%, Sp 82%)	-Portable device -Immediate assessment of steatosis -Simultaneous liver stiffness measurement	-High rate of measurement failure particularly in obese patients
MRI-PDFF	-Option that can be added to MRI scanners to quantitatively assess steatosis	AUROC 0.98 (Sn 93%, Sp 94%)	-Not affected by obesity -Simultaneous MRI for liver architecture and focal lesions	-Time-consuming -Requires MRI facility -Costly -Cannot be used in some patients with implantable devices
^1^H-MRS	-Provides a collection of spectra for signal fat fraction estimation, which requires a proper acquisition technique to estimate hepatic triglyceride content	AUROC 0.98 (Sn 89%, Sp 92%)	-The absolute hepatic triglyceride concentration can be directly measured, and very small amounts (as low as 0.5%) can be detected and quantified	-See MRI-PDFF -Complex and time-consuming data analysis

Abbreviations: *AUROC*, area under the receiver operating characteristic; *MRI-PDFF*, magnetic resonance imaging-proton density fat fraction; ^*1*^*H-MRS*, proton magnetic resonance spectroscopy; *Sn*, sensitivity; *Sp*, specificity

## Fibrosis

Detecting fibrosis in NAFLD patients is critical as advanced fibrosis (F3-F4) independently predicts the development of liver-related complications, the need for liver transplantation, and liver-related and overall mortality [16–19]. Advanced fibrosis is also associated with a higher incidence of chronic kidney disease and increased mortality from cardiovascular disease [74]. Thus, there is a need to identify patients with bridging fibrosis (F3) and cirrhosis (F4) so that they can be managed to delay further progression, particularly given a large number of patients with undiagnosed cirrhosis within the general population (6–7%) [75].

Given the limitations of biopsy for widescale screening, NITs for assessment of fibrosis stage will play an increasing role in the management of NAFLD patients. The two main types of NITs used are the following: predictive models, which use clinical and laboratory data and imaging techniques, which estimate liver stiffness as a potential surrogate of hepatic fibrosis.

### Serum-based fibrosis biomarkers

The most common approach to assessing the stage of fibrosis by serological means consists of routine biochemical and/or hematological tests. These are indirect serum markers and are based on the evaluation of common functional alterations in the liver, alterations that do not necessarily reflect extracellular matrix turnover and/or fibrogenic cell changes. A better understanding of the pathophysiology of liver fibrosis has prompted investigators to use more refined markers to identify different fibrosis stages. These so-called direct serum markers are intended to detect extracellular matrix turnover and/or fibrogenic cell changes. Markers may be used alone or combined with patient and clinical characteristics or with other direct or indirect markers to form panels. An overview of the most common combinations of non-invasive serum-based biomarkers is presented in Table 3.

**Table 3:** Serum-based biomarkers of liver fibrosis in NAFLD

Algorithm	Formula/parameters	Number of NAFLD patients	AUROC/Sn/Sp/NPV/PPV
APRI[88]	(AST (U/L)/(AST upper limit of normal))/(platelet count (× 10^9^/L) × 100)	145	0.67/27/89/84/37
FIB-4[88–90]	(age (years) × AST (U/L)) / ((platelet count (× 10^9^/L)) × (ALT(U/L))^1/2^)	145 541 1038	0.86/85/65/95/36 0.80/52/90/–/– 0.85/84/69/–/–
NFS[88–90]	− 1.675 + 0.037 × age (years) + 0.094 × BMI (kg/m^2^) + 1.13 × impaired fasting glucose/diabetes (yes = 1, no = 0) + 0.99 × AST/ALT ratio – 0.013 × platelet count (× 10^9^/L) – 0.66 × albumin (g/dL)	733 145 1038	0.82–0.88/77–82/71–77/88–93/52–56
BARD[89, 91, 92]	BMI ≥ 28: No = 0, Yes = 1; AST/ALT ratio ≥ 0.8: No = 0, Yes = 2; and diabetes: No = 0, Yes = 1	827 145 138 1038	0.81/–/–/–/– 0.77/89/44/95/27 0.67/51/77/81/45 0.76/74/66/–/–
ELF[93, 94]	2.2781 + 0.851 × ln [HA] (µg/L) + 10.751 × ln [P3NP] (µg/L) + 10.934 × ln [TIMP 1] (µg/L)	61 192	0.87/89/96/96/80 0.90/80/90/94/71
FibroMeter[95]	Platelets, prothrombin index, AST, α2-macroglobulin, hyaluronic acid, urea, age	383	0.89*/81*/84*/77*/86*
FibroTest[96]	Haptoglobin, α2-macroglobulin, apolipoprotein A1, GGT, bilirubin, age, gender	267	0.81/92/71/98/33

^*^Values are for prediction of significant fibrosis. *ALT*, alanine aminotransferase; *APRI*, AST-to-platelet ratio index; *AST*, aspartate aminotransferase; *AUROC*, area under the receiver operating characteristic; *BMI*, body mass index; *DM*, diabetes mellitus; *ELF*, enhanced liver fibrosis; *FIB-4*, fibrosis-4 score; *GGT*, gamma glutamyltransferase; *IFG*, impaired fasting glycemia; *MMP*, matrix metalloproteinases; *NAFLD*, non-alcoholic fatty liver disease; *NFS*, non-alcoholic fatty liver disease fibrosis score; *NPV*, negative predictive value; *P3NP*, N-terminal peptide of procollagen III; *PPV*, positive predictive value; *TIMP*, tissue inhibitors of metalloproteinases

Fibrosis-4 (FIB-4) and NAFLD fibrosis score (NFS) are the most widely used algorithms and can be easily calculated using freely available online calculators [76, 77]. Both algorithms have high negative predictive values (approximately 90%) and can reliably exclude advanced fibrosis, thus identifying lower-risk patients who do not need secondary care referral. However, although FIB-4 and NFS are effective at excluding advanced fibrosis, as well as predictive of liver outcomes over time [78], they have limited ability to identify NAFLD patients with advanced fibrosis. Approximately 30% of NAFLD patients will be classified as “indeterminate” when using FIB-4 and NFS, and a significant number are falsely classified as having advanced fibrosis. Since there is abundant evidence that NFS and FIB-4 provide higher rates of false-positive results in older populations, it has been proposed that different diagnostic cut-offs should be used in patients ≥ 65 years old [79]. However, the latter proposal has been criticized since a recent study suggested that these adapted cut-offs significantly decreased the sensitivity to only 60% in patients over 65 years of age [80]. It should be noted that FIB-4 and NFS have been developed and, therefore, their coefficients for age have been calibrated in cohorts of patients aged around 45–50 years. Consequently, they are less sensitive in younger people and should not be used in patients < 35 years old [81]. This could become a matter of importance if cases of advanced liver fibrosis in young adults increase as expected due to the dramatic increase in the prevalence of obesity and insulin resistance in children [82].

Commercial biomarker panels such as the enhanced liver fibrosis (ELF) test, FibroTest (FibroSure), and FibroMeter have been developed, though they are more expensive and less available than FIB-4 and NFS. ELF is a simplified algorithm comprising three direct fibrosis markers which, aside from distinguishing patients with advanced fibrosis, may also be a good predictor of liver-related morbidity and mortality [82]. Therefore, this test may be considered in patients where FIB-4 and NFS indicate advanced fibrosis.

Several new scoring systems have also reported good accuracy in detecting advanced fibrosis in patients with NASH. Among them, an algorithm based on the measurement of serum PRO-C3 (a marker of type III collagen formation), age, presence of diabetes, and platelet count (ADAPT) [83], and the LINKI algorithms, which are based on hyaluronic acid, fasting glucose, AST, age, and platelet count [84]. In addition, the Hepamet fibrosis score has recently demonstrated superior diagnostic accuracy, compared to FIB-4 and NFS, in a multinational cohort of 1500 patients [85, 86]. Recently, a Cox regression model based on age, AST/ALT-ratio, and ALT-level (dAAR) was reported to predict the risk of incident severe liver outcomes in the general population and may be of value for detecting advanced liver fibrosis [87].

### Ultrasound-based methods

Techniques based on elastography measure tissue stiffness as a physical property termed Young’s modulus [97]. VCTE measures the speed of a mechanically induced shear wave through the hepatic parenchyma using pulse-echo ultrasonic acquisitions to obtain a liver stiffness measurement (LSM) as a marker of hepatic fibrosis. The velocity of the shear wave, which is perpendicular to the direction of pulse wave propagation, is proportional to liver stiffness, with quantitative results available as the algebraically derived Young’s modulus in kilopascals [97]. The result of VCTE is obtained as a median of at least 10 measurements and measures approximately a 1-cm diameter by 4-cm length region of liver tissue, which is approximately 100 times larger than that evaluated with liver biopsy [97]. VCTE is an easy-to-perform tool using a portable ultrasound probe, either an M-probe (3.5 MHz, at 2.5 to 6.5 cm depth) or, in the case of morbid obesity, an XL-probe (2.5 MHz, at 3.5 to 7.5 cm depth). Values of LSM < 8 kPa exclude advanced fibrosis with a very high probability [98]. However, the ability to rule in advanced fibrosis is considerably lower. Cut-offs ranging from 8 to 12 kPa have been proposed, with sensitivities ranging from 84 to 100% and specificities from 83 to 97% [48].

Obesity increases the probability of invalid results (by up to 30%) and may also lead to an overestimation of LSM [99, 100]. With the availability of the XL probe, which has been approved for use in patients with morbid obesity, the failure rate of VCTE was reported to be < 5%. VCTE data are not reliable under conditions in which a rapidly developing mass effect inside the liver increases intrahepatic pressure and, thereby, reduces liver elasticity. This phenomenon is observed in right-sided congestive heart failure, acute inflammation and/or edema of the liver, and extrahepatic cholestasis [48]. In addition, regarding food intake, a minimum 2-h fast is currently recommended prior to the examination [48].

Shear wave elastography (SWE) and acoustic radiation force impulse (ARFI) are integrated into conventional ultrasonography devices to measure LSM [101]. Although SWE has been reported to perform better than ARFI in differentiating moderate fibrosis in NAFLD patients, VCTE, SWE, and ARFI showed similar performances in differentiating advanced fibrosis and suffered from a significant rate of failures or unreliable results [102].

### Magnetic resonance-based methods

**Fig. 3:**
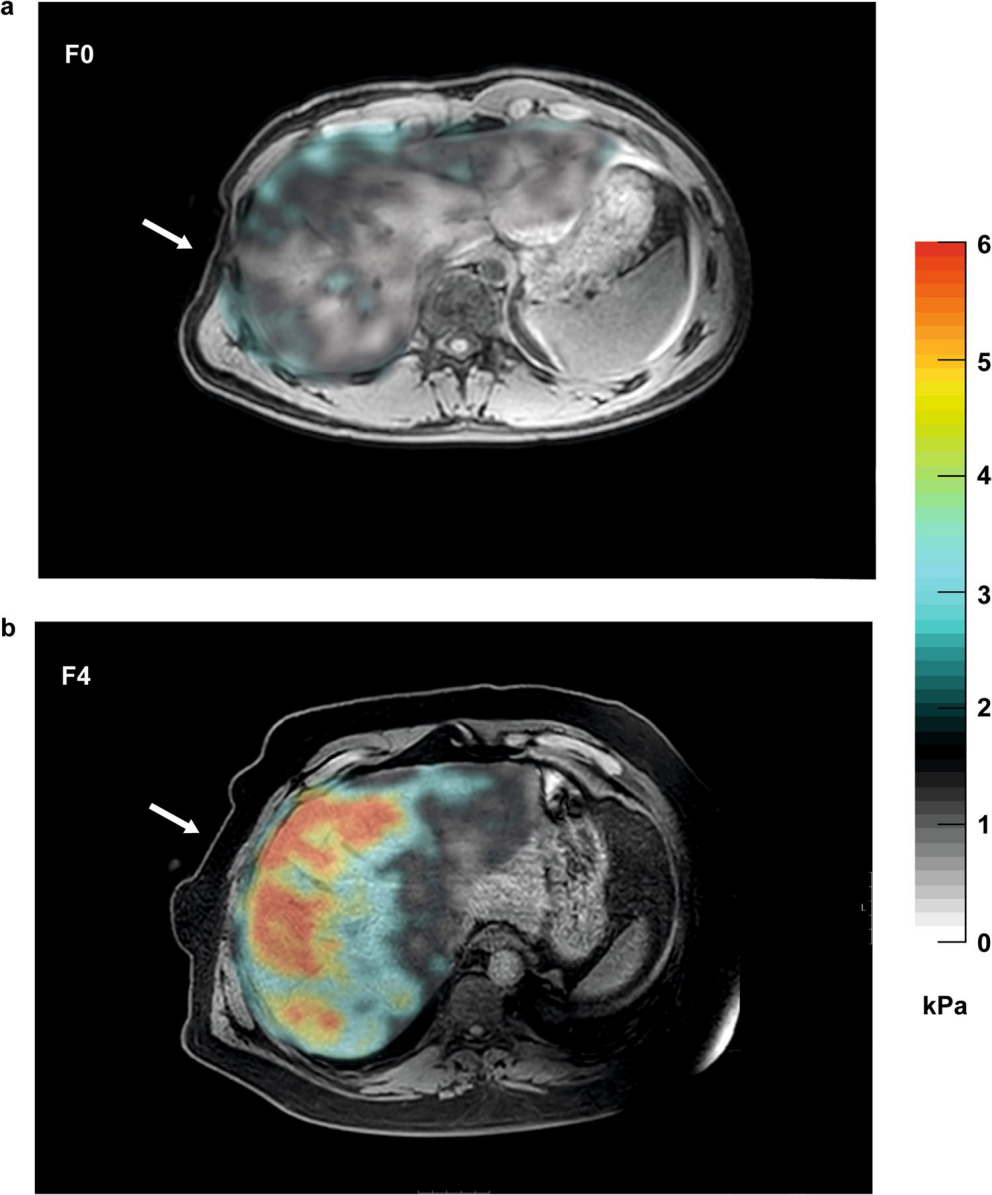
Hepatic magnetic resonance elastography (MRE) images captured from two NAFLD patients using an active electrodynamic transducer transmitting mechanical waves at 54 Hz. Arrow indicates placement of transducer when strapped to the patient. Tissue stiffness is illustrated in a corresponding elastogram, shown as a color gradient with higher degrees of tissue stiffness indicated in red. Liver biopsies obtained immediately after the MRE-examination showed **a** absence of fibrosis (F0) and **b** cirrhosis (F4), respectively. Abbreviations: kPa, kilopascal; NAFLD, non-alcoholic fatty liver disease

With magnetic resonance elastography (MRE), low-frequency vibrations are applied to the abdominal wall, which are tracked as propagating hepatic shear waves by acquiring images with wave motion-sensitized phase-contrast sequences and processing raw images (i.e., magnitude and phase images) to “wave images” and then to “elastogram” (Fig. 3). The cross-sectional elastogram images reflect the stiffness generated from the wave propagation information [103]. An MRE protocol can be performed with most MR scanners but requires adding hardware to generate mechanical waves and software for acquisition and processing. In theory, the whole liver can be examined, but typically four axial (or transverse) slices with a thickness of 5–10 mm are placed in the widest part of the liver, which generally corresponds to 5–35% of the total liver volume. Liver stiffness measurement is performed by drawing a region of interest on the generated elastograms, avoiding edge effects, large vessels, the gallbladder fossa, and any areas affected by cardiac and vascular artifacts. The mean stiffness is calculated using the mean from all regions of interest. Thus, MRE evaluates much larger volumes of the total liver than liver biopsy and can be performed in conjunction with conventional MRI.

Shear stiffness values in the normal liver are < 3 kPa [103]. Studies have demonstrated that MRE has high diagnostic accuracy, with an AUROC of ≥ 0.90 for identifying advanced fibrosis in NAFLD with MRE cut-off values ranging from 3.64 to 4.15 kPa [67, 104–106]. As with VCTE, MRE needs to be performed in the fasting state, at least for 2 h, because increased postprandial portal blood flow may cause a dynamic increase in liver stiffness, potentially leading to an overestimation of the extent of fibrosis [103]. In contrast to VCTE, diagnostic accuracy is not affected by patient body factors. In addition, the inter-observer agreement for staging fibrosis is nearly perfect and higher than that seen with histopathology [67, 105, 106].

The performance of MRE is better than that of VCTE-LSM in diagnosing advanced fibrosis in NAFLD patients [66]. However, unlike VCTE, it is difficult to implement MRE at a large scale since it requires an MRI facility, is associated with a high cost, and is time-consuming.

## Evolving non-invasive tests

### Genetic markers

Genome-wide association studies have revealed several genetic risk factors associated with NAFLD prevalence and progression. Moreover, functional studies on those genes have added much knowledge on the pathogenesis of NAFLD and the complex pathophysiologic pathways related to the disease [107]. The risk variant with the most pronounced effect on the development and progression of NAFLD is the rs738409 C > G single nucleotide polymorphism of patatin-like phospholipase domain-containing 3 (*PNPLA3)*, which encodes the I148M protein variant of PNPLA3, also known as adiponutrin, a protein involved in lipid remodeling [108]. The primary hepatic effects of the I148M protein variant are most likely attributable to its repression of lipase activity [109], resulting in increased intracellular accumulation of triglycerides with the number of risk alleles (the G allele). In a recent study in which subjects were phenotyped by MRI-PDFF, each copy of the PNPLA3 risk allele was associated with a 2.56% increase in hepatic steatosis [110]. In addition, cumulative evidence also suggests that *PNPLA3* is associated with fibrosis progression, development of HCC, ESLD, and all-cause mortality [111].

The gene encoding transmembrane 6 superfamily member 2 (*TM6SF2*) is also associated with NAFLD progression. The gene product of *TM6SF2* is involved in VLDL secretion from hepatocytes, and the *TM6SF2* E167K variant results in a loss of this function, resulting in the hepatic accumulation of lipids [112]. In NAFLD patients, *TM6SF2* E167K is associated with significant fibrosis (stages 2–4), with an odds ratio (OR) of 1.88, independent of the *PNPLA3* genotype [113].

Glucokinase regulatory protein (GCKR) is an inhibitor of glucokinase and regulates de novo lipogenesis [114]. The *GCKR*P446L variant disrupts negative regulation of glucokinase, which leads to glucose uptake and increased de novo lipogenesis. In a study comparing patients with NAFLD with healthy control individuals, significant associations were found between *GCKR* rs780094 and susceptibility to NAFLD (OR 1.49), NASH (OR, 1.55), and NASH with significant fibrosis (OR, 1.50) [115]. High levels of liver fat caused by these genetic mutations seem to be associated with disease progression in NAFLD. Thus, due to their influence on hepatic lipid accumulation, these genetic risk variants further support the hypothesis that high levels of liver fat are associated with the progression of NAFLD and probably other chronic liver diseases as well.

Interestingly, several genes protective of hepatic steatosis and fibrosis have recently been reported, namely, mitochondrial amidoxime-reducing component 1 (MARC1), 17β-hydroxysteroid dehydrogenase 13 (HSD17B13), LPIN1, uncoupling protein 2 (UCP2), interleukin-28B (IL-28B), Kruppel-like factor 6 (KLF6), and the MER protocol-oncogene tyrosine kinase (MERTK) [111].

### Omics-based markers

Through their ability to detect thousands of different molecules, “omics technologies” represent a new diagnostic approach that could be useful for the diagnosis of hepatic steatosis. A proteomics study conducted on 70 patients (35 controls and 35 NAFLD) identified 20 protein peaks in NAFLD versus controls (sensitivity of 89% and specificity of 83%). Moreover, it was reported that NAFLD patients had a higher basal hemoglobin level [116]. Metabolomics has been applied to identify the metabolic profile specific for NAFLD, such as glutathione and bile acids, whose levels are altered during NAFLD onset [117]. Lipidomic-based studies identify the alteration of lipid species levels, for instance, eicosanoids and short-chain fatty acids. It has been reported that three specific lipids (defined as “lipid triplet”) can identify NAFLD. However, the diagnostic performance was modest (AUROC of 0.71–0.74) [118]. Omics-based biomarkers may also be of value to evaluate disease severity. It was recently reported that a signature of triglycerides was able to differentiate steatosis from NASH, however, with limited diagnostic performance (AUROC of 0.79) [119].

### RNA biomarkers

In biological fluids, there are RNA molecules belonging to different classes. A key feature making these RNAs potentially valuable biomarkers is their high stability since they are not present in free form in the circulation. Instead, they are encapsulated in membranous vesicles, complexed to RNA-binding proteins, or associated with lipoproteins, features that protect these RNAs from degradation [120]. Moreover, the techniques used for their detection are extremely sensitive. In theory, even a single RNA molecule could be detected through quantitative PCR [121].

Most of the studies concerning circulating RNAs as NAFLD biomarkers are limited to microRNAs (miRNAs). It has been reported that a miRNA panel (miR-122, miR-1290, miR-27b, miR-192) showed a high NAFLD diagnostic accuracy (AUROC of 0.86) [122]. Moreover, in a recent study, 2083 serum miRNAs were profiled in NAFLD patients representing the complete NAFLD spectrum and compared with population controls. The most robust finding was that serum miR-193a-5p levels correlated strongly with NAFLD activity grade and fibrosis stage. It was concluded that miR-193a-5p is a potential clinically tractable circulating biomarker for progressive NAFLD [123].

### Gut microbiota

Several hypotheses have provided mechanistic insights into the pathways of how the gut microbiota might contribute to NAFLD development and progression [124]. Some studies have focused on microbiome signatures in NAFLD with fibrosis of different stages. When compared with NAFLD patients with severe fibrosis, individuals with absence of or mild fibrosis as well as healthy controls display a decreased abundance of Gram-negative bacteria, decreased Fusobacteria phylum, increased Enterobacteriaceae family (*Bacteroides*, *Ruminococcus*, and *Shigella* genera [125, 126]), and, by contrast, increased Gram-positive bacteria, Firmicutes phylum, Prevotellaceae family, and *Prevotella* genus [127].

In a recent study, Lang et al. used gut microbiota-based approaches, VCTE, NFS, and FIB-4, to predict advanced fibrosis in 83 biopsy-proven NAFLD patients. A random forest model composed of clinical features and bacterial taxa achieved an AUROC of 0.87, which was similar to the AUROCs of NFS and FIB-4 (0.86 and 0.85, respectively). VCTE had the best diagnostic performance with an AUROC of 0.93 [128].

## Clinical implications

### Which patient populations should be screened for NAFLD?

Current guidelines do not recommend widespread or community screening, mainly due to the perceived associated direct and indirect medical costs [20, 39]. However, some national and international initiatives (ETHON project, Spain [129]; international LiverScreen project [NCT03789825]) are currently investigating the effectiveness of screening the general population for significant liver disease. Screening at-risk patients has been shown to be cost-effective in several studies across different countries [130–132]. To define at-risk populations, important information can be obtained from population-based studies evaluating the screening for liver fibrosis using VCTE [81, 133–135]. Using the 8.0 kPa threshold, the prevalence of patients at risk of significant liver fibrosis among adults in the general population was similar across studies (6–7%). However, in some subgroups, the prevalence was significantly higher. T2DM has been shown to be the condition associated with the highest risk of increased LSM [133]. The prevalence of liver stiffness ≥ 8.0 kPa was approximately 9% in patients with diabetes but no liver steatosis, and it reached 17.2% in patients with both diabetes and liver steatosis [134].

Other factors associated with elevated liver stiffness are closely related to metabolic conditions such as obesity, impaired fasting glucose, low HDL-cholesterol, and high triglyceride levels. [8, 136–138]. The available evidence, therefore, suggests that patients with BMI > 25 kg/m^2^, particularly those with metabolic risk factors, and especially T2DM, might be a relevant population for the identification of NAFLD patients with advanced fibrosis. However, it is unclear at which age at-risk patients should be screened. Most studies have been performed in adults aged > 40 years, although obesity and T2DM have become more prevalent in children. Moreover, T2DM tends to be more aggressive in young people with poorer responses to glucose-lowering medication and greater insulin resistance [139–141]. As the length of exposure to insulin resistance is probably a key factor in the development of NAFLD with advanced fibrosis, it is reasonable to assume that more cases of advanced NAFLD will be identified in young adults in the near future. In this context, it was recently reported that the prevalence of elevated LSM (≥ 7.9 kPa) in young adults aged 22–25 years was 2.7% [81].

### Which method should be used to identify steatosis?

As stated above, ^1^H-MRS and MRI-PDFF can be regarded as reference methods to diagnose steatosis. These methods provide a more accurate assessment of steatosis than conventional histopathology, which often overestimates hepatic triglyceride content [24, 71]. However, despite the high accuracy of MRI-PDFF for detecting and quantifying steatosis, cost and limited availability restrict its use on a larger scale.

Serum-based algorithms to identify steatosis have been proposed for screening purposes, but, in general, their diagnostic performance is suboptimal compared to other methods, and currently, they do not add much to the information provided by the clinical, laboratory, and imaging examinations that are routinely performed in patients with suspected NAFLD.

Conventional ultrasonography is currently recommended as the first-line tool for the diagnosis of steatosis in clinical practice [39]. It is widely available, cheap, and well-established. However, ultrasonography has poor sensitivity for detecting steatosis < 20% and many NAFLD patients may thus be missed when using this modality. CAP is a promising technique for the detection of steatosis, but it is unknown whether its diagnostic accuracy is better than that of ultrasonography in patients with steatosis < 20%. If NAFLD patients with steatosis missed by ultrasound-based techniques have a low risk of developing advanced fibrosis, the inability of conventional ultrasonography and CAP to identify steatosis < 20% would be of limited clinical importance. However, a phenomenon exists in NAFLD known as burned-out NASH, in which steatosis decreases with the progression of fibrosis [142]. In a study including 458 NAFLD patients with advanced fibrosis, overall mortality was higher in patients with histological steatosis < 33% than in those with steatosis ≥ 33% [143]. Thus, some NAFLD patients with a low steatosis grade not identified with ultrasound-based methods might have advanced fibrosis and an increased risk of liver-related events and mortality. The characteristics of NAFLD patients diagnosed with MRI-PDFF but missed by ultrasound-based techniques will be elucidated in the currently ongoing prospective Swedish EPSONIP study [144] in which patients with T2DM consecutively enrolled from primary care health centers will undergo serological, ultrasound-based, and magnetic resonance-based repeated measurements of steatosis and fibrosis.

### Which method should be used to identify advanced fibrosis in NAFLD?

**Fig. 4:**
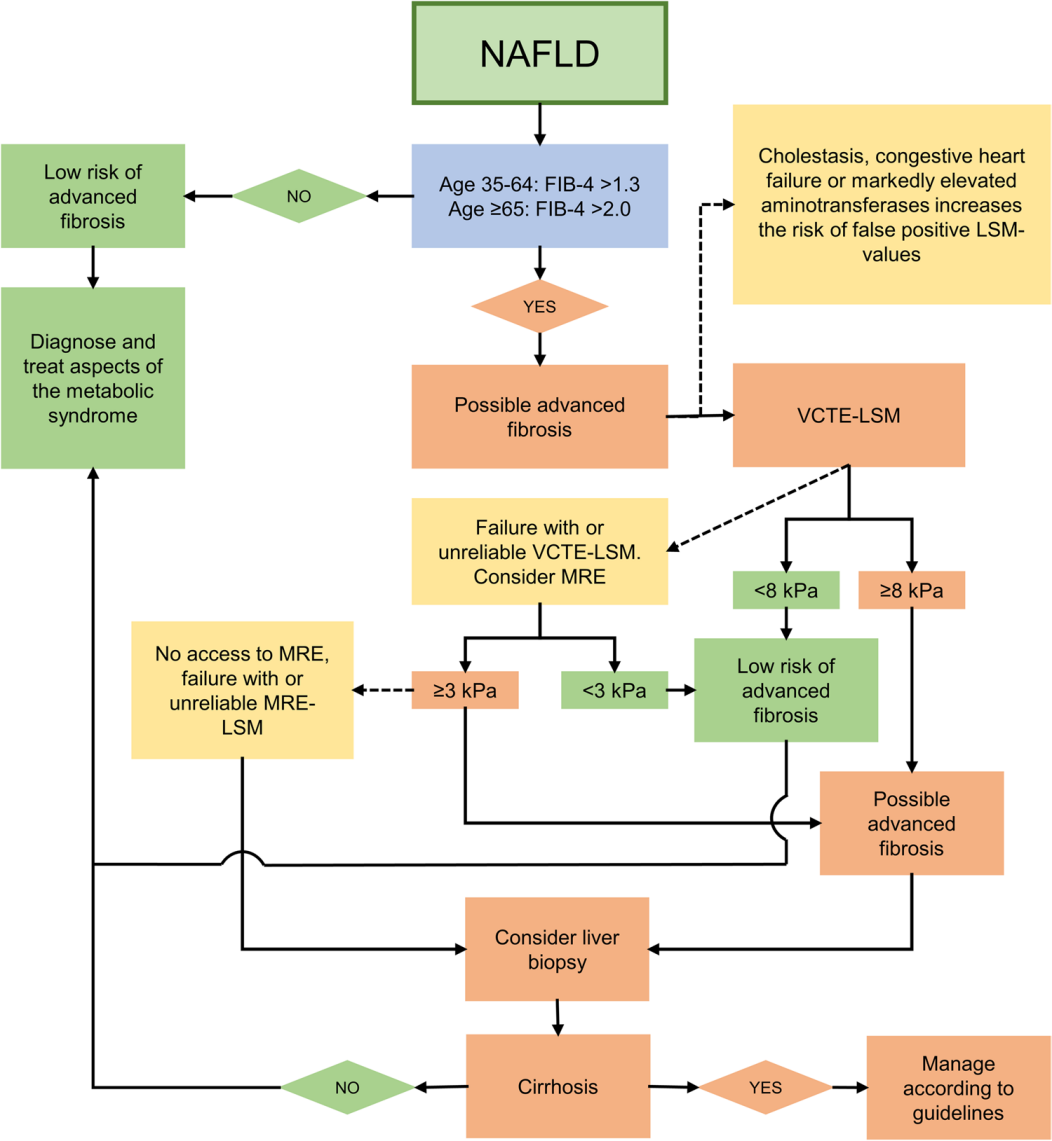
Suggested algorithm for non-invasive risk stratification of NAFLD patients without signs of end-stage liver disease. Abbreviations: FIB-4, fibrosis-4; kPa, kilopascal; LFT, liver function test; LSM, liver stiffness measurement; MRE, magnetic resonance elastography; NAFLD, non-alcoholic fatty liver disease; VCTE, vibration-controlled transient elastography

The most validated NITs for assessment of fibrosis stage in NAFLD are NFS and FIB-4, which are non-patented and widely available tests. FIB-4 is easier to calculate since it is composed of only three serum-based parameters and the subject’s age, and thus may be most suitable for screening purposes. Both algorithms can exclude the presence of advanced fibrosis with a high NPV (> 90%) [145]. However, their PPV for confirming advanced fibrosis is modest (< 70%), with a significant risk of false-positive results [145]. Moreover, approximately one-third of patients fall in between the lower and upper cut-offs, resulting in undetermined results [145]. The insufficient PPV of these tests has led to the development of strategies using FIB-4 or NFS as a first-line procedure, followed, if positive, by a second-line confirmatory test (elastography or specialized blood test). A proposed pathway to assess the fibrosis stage in NAFLD patients is shown in Fig. 4.

The two most validated patented serum fibrosis biomarkers are FibroMeter and ELF. Overall, the diagnostic accuracy of patented serum fibrosis tests for staging fibrosis is similar [146] to that of FIB-4 and NFS or only slightly higher [147]. However, high costs and limited availability limit the widespread application of these tests in clinical practice.

VCTE is the most widely available device for LSM with the largest amount of data in the NAFLD setting. It has significantly better diagnostic accuracy compared to FIB-4 and NFS [145] and is, therefore, better suited as a second-line test. There is no agreement in clinical practice on LSM cut-offs for ruling out advanced fibrosis, even though 8 kPa is the most validated threshold, with an NPV > 90%. However, the PPV to confirm advanced fibrosis is not sufficiently high to avoid liver biopsies in many NAFLD patients. According to the results of a recent meta-analysis [98], values of LSM by VCTE > 12 kPa could be used to rule in advanced fibrosis, thus limiting the need for confirmatory liver biopsy in NAFLD patients with LSM values by VCTE between 8 and 12 kPa, albeit this must be confirmed in further studies. Regarding SWE, meta-analyses [148, 149] suggest performance in detecting advanced fibrosis similar to that reported for VCTE [102]. However, SWE is less available and relevant data in NAFLD patients remain limited.

MRE has superior diagnostic accuracy compared to ultrasound-based elastography techniques. However, the use of MRE is limited to tertiary specialized centers because of its high cost and the need for advanced equipment.

### Should screening aim to identify steatosis, advanced fibrosis, or earlier stages of fibrosis?

In light of the pathophysiology of NAFLD, which implies the initial accumulation of triglycerides leading to steatosis and, in some individuals, subsequent progressive fibrosis, it is reasonable to screen for steatosis in patients with metabolic risk factors such as T2DM and hyperlipidemia, or with BMI > 25 kg/m^2^. Considering that high levels of liver fat appear to be associated with the progression of NAFLD, ultrasonography, which is widely available and has sufficient sensitivity to identify pronounced steatosis, is currently the screening method of choice. Further investigations can be avoided in subjects without steatosis, while in those with steatosis, an NIT, preferably FIB-4, should be used to assess the probability of advanced fibrosis (F3-F4). If positive, a second-line confirmatory test (elastography or specialized blood test) should be conducted.

However, applying the strategy above theoretically entails a risk of missing advanced fibrosis in burned-out NASH or in NAFLD patients with low steatosis grade not detectable with ultrasonography. Therefore, an alternative screening approach may be to disregard steatosis and focus on screening for advanced fibrosis with serum-based NITs and/or elastography in populations at risk for NAFLD. Future studies in subjects with metabolic risk factors assessing the prevalence of advanced fibrosis with normal HTGC or with low steatosis grade undetectable with ultrasonography, as well as the diagnostic performance of NITs in these patients, will elucidate whether this strategy may be preferable.

Identifying earlier stages of fibrosis in NAFLD is appealing since this would give physicians opportunities to intervene at an earlier stage, minimizing the risk of future liver-related complications. However, considering the inferior diagnostic performance of NITs to distinguish fibrosis stages 1–2 and in light of the absence of approved pharmacological treatments to halt or retard disease progression, it is currently advisable to focus on identifying NAFLD patients with advanced fibrosis. The presence of advanced fibrosis, particularly cirrhosis, alters clinical management**,** especially the initiation of surveillance for gastroesophageal varices and HCC.

## Conclusions

The increasing prevalence of obesity has changed the landscape of chronic liver disease, with increasing numbers of NAFLD patients presenting with complications of ESLD. In many of these patients, NAFLD and its severity have previously been undiagnosed or neglected, and opportunities for early interventions and surveillance have been missed. Accumulating evidence supports the use of NITs for diagnosing steatosis and assessing the presence of advanced fibrosis in NAFLD. The diagnostic performance of serum-based algorithms is currently insufficient for clinical use to identify patients with steatosis. CAP and other ultrasound-based methods are promising, but diagnostic cut-offs for steatosis are yet to be defined. ^1^H-MRS or MRI-PDFF can reliably diagnose steatosis and replace liver biopsy as the reference method in this aspect. On the other hand, their limited availability limits the implementation of these methods on a large scale. Serum-based algorithms are recommended as the first step to assess the fibrosis stage in NAFLD. If advanced fibrosis cannot be excluded with these algorithms, VCTE offers higher diagnostic accuracy with excellent NPV but with considerably lower PPV. Thus, liver biopsy may still be needed to assess the fibrosis stage in a significant number of NAFLD patients. MRE may reduce the need for liver biopsy, although further studies are needed to clarify its usefulness in the evaluation and follow-up of NAFLD patients. Genetic, omics-based, and RNA markers, as well as gut microbiota-based approaches are promising methods, but their role in the management of NAFLD patients remains to be clarified.

## Abbreviations

ALT: Alanine aminotransferase
ARFI: Acoustic radiation force impulse
AST: Aspartate aminotransferase
ATI: Attenuation imaging
ATT: Attenuation coefficient
AUROC: Area under the receiver operating characteristic
BMI: Body mass index
CAP: Controlled attenuation parameter
CT: Computed tomography
ELF: Enhanced Liver Fibrosis
ESLD: End-stage liver disease
FLI: Fatty liver index
FIB-4: Fibrosis-4
GCKR: Glucokinase regulatory protein
GGT: Gamma glutamyltransferase
HCC: Hepatocellular carcinoma
HDL: High-density lipoprotein
^1^H-MRS: Proton magnetic resonance spectroscopy
HSD17B13: 17β-Hydroxysteroid dehydrogenase 13
HSI: Hepatic steatosis index
HTGC: Hepatic triglyceride content
HU: Hounsfield unit
IL-28B: Interleukin-28B
KLF6: Kruppel-like factor 6
LAP: Lipid accumulation product
LSM: Liver stiffness measurement
MARC1: Mitochondrial amidoxime-reducing component 1
MAFLD: Metabolic dysfunction-associated fatty liver disease
MERTK: MER protocol-oncogene tyrosine kinase
MRE: Magnetic resonance elastography
MRI: Magnetic resonance imaging
MRI-PDFF: Magnetic resonance imaging proton density fat fraction
NAFLD: Non-alcoholic fatty liver disease
NAFLD-LFS: NAFLD liver fat score
NAS: NAFLD activity score
NASH: Non-alcoholic steatohepatitis
NFS: NAFLD fibrosis score
NIT: Non-invasive test
NPV: Negative predictive value
OR: Odds ratio
PNPLA3: Patatin-like phospholipase domain-containing 3
PPV: Positive predictive value
Sn: Sensitivity
Sp: Specificity
SPC: Stereological point counting
SWE: Shear wave elastography
T2DM: Type 2 diabetes mellitus
TG: Triglycerides
TyG: Triglyceride x glucose
TM6SF2: Transmembrane 6 superfamily member 2
UCP2: Uncoupling protein 2
UGAP: Ultrasound-guided attenuation parameter
ULN: Upper limit of normal
VAI: Visceral adiposity index
VCTE: Vibration controlled transient elastography
VLDL: Very low-density lipoprotein
WC: Waist circumference
